# Supplementary material for: Screening of phytoconstituents from Bacopa monnieri (L.) Pennell and Mucuna pruriens (L.) DC. to identify potential inhibitors against Cerebroside sulfotransferase
Source: PLoS One. 2024 Oct 24;19(10):e0307374. doi: 10.1371/journal.pone.0307374 (PMC11500956; doi:10.1371/journal.pone.0307374)
Supplement: S1 File — (DOCX) [file pone.0307374.s001.docx]

**Supplementary information**

**Screening of phytoconstituents from *Bacopa monnieri* *(L.) Pennell* and *Mucuna pruriens (L.) DC.* to identify potential inhibitors against *Cerebroside Sulfotransferase*.**

**Nivedita Singh^a^* and Anil Kumar Singh^a^**

^a^Department of Dravyaguna, Faculty of Ayurveda, Institute of Medical Sciences, Banaras Hindu University, Varanasi, Uttar Pradesh-221005, India

*Corresponding Author

Dr. Nivedita Singh, Department of Dravyaguna, Faculty of Ayurveda, Institute of Medical Sciences, Banaras Hindu University, Varanasi, Uttar Pradesh-221005, India-221005, Email: niv234@gmail.com, s.nivedita@bhu.ac.in

**S1 Table.** **List of 35 phytoconstituents of** ***Bacopa monnieri* with their binding scores in the largest cluster of protein-ligand complex**

| **Sl. No.** | **IMMPAT ID** | **Phytochemical Name** | **Binding score in largest cluster** | **Number of conformations in largest cluster** |
| --- | --- | --- | --- | --- |
|  | IMPHY000309 | Dotriacontane | -2.82 | 18 |
|  | IMPHY001534 | Jujubogenin | -8.24 | 53 |
|  | IMPHY001896 | Heptacosane | -2.85 | 25 |
|  | IMPHY002343 | Bacogenin a2 | -8.75 | 17 |
|  | IMPHY002389 | Bacogenin-a1 | -8.03 | 49 |
|  | IMPHY002708 | Pseudojujubogenin | -8.14 | 93 |
|  | IMPHY004187 | L-(+)-Arabinose | -3.78 | 23 |
|  | IMPHY004660 | Luteolin | -5.52 | 26 |
|  | IMPHY004661 | Apigenin | -5.94 | 28 |
|  | IMPHY005478 | Strychnine | -6.60 | 50 |
|  | IMPHY006362 | Ascorbic acid | -4.56 | 22 |
|  | IMPHY006699 | Bacosine | -8.19 | 56 |
|  | IMPHY007301 | Nicotine | -4.36 | 66 |
|  | IMPHY007325 | Plantainoside B | -5.70 | 3 |
|  | IMPHY007357 | Nicotinic acid | -6.17 | 58 |
|  | IMPHY007395 | Bacoside A3 | -5.47 | 15 |
|  | IMPHY007882 | Ebelin lactone | -8.68 | 46 |
|  | IMPHY008910 | Hentriacontane | -3.54 | 16 |
|  | IMPHY009413 | Triacontane | -3.07 | 29 |
|  | IMPHY009481 | Octacosane | -3.45 | 28 |
|  | IMPHY009482 | Nonacosane | -3.82 | 21 |
|  | IMPHY009532 | 4-Hydroxy-2H-pyran-3-carboxaldehyde | -4.29 | 52 |
|  | IMPHY009537 | Stigmastenol | -9.02 | 45 |
|  | IMPHY011646 | Cynaroside | -6.76 | 11 |
|  | IMPHY011710 | Apigenin-7-o-glucuronide | -7.05 | 13 |
|  | IMPHY011711 | Apigenin 7-glucuronide | -8.72 | 26 |
|  | IMPHY011729 | Mannitol | -1.59 | 11 |
|  | IMPHY012003 | Betulinic acid | -9.27 | 52 |
|  | IMPHY013594 | Bacoside B | -7.10 | 28 |
|  | IMPHY014827 | Bacoside A | -5.75 | 10 |
|  | IMPHY014836 | beta-Sitosterol | -8.64 | 53 |
|  | IMPHY014842 | Stigmasterol | -8.65 | 31 |
|  | IMPHY014893 | D-Glucose | -4.45 | 65 |
|  | IMPHY014899 | Stigmastanol | -8.57 | 38 |
|  | IMPHY015494 | Bacopaside I | -4.53 | 5 |

**S2 Table.** **List of 31 phytoconstituents of** ***Mucuna pruriens* with their binding scores in the largest cluster of protein-ligand complex**

| **Sl. No.** | **IMMPAT ID** | **Phytochemical Name** | **Binding score in largest cluster** | **Number of conformations in largest cluster** |
| --- | --- | --- | --- | --- |
|  | IMPHY000060 | Myristic acid | -5.51 | 50 |
|  | IMPHY001252 | Bufotenine | -5.03 | 73 |
|  | IMPHY001842 | Tryptamine | -5.31 | 60 |
|  | IMPHY003370 | N,N-Dimethyl-5-methoxytryptamine | -4.61 | 50 |
|  | IMPHY003490 | Coumarin | -6.07 | 47 |
|  | IMPHY004055 | Choline | -2.87 | 43 |
|  | IMPHY004141 | alpha-Amyrenyl acetate | -8.92 | 58 |
|  | IMPHY004611 | Acacetin | -5.66 | 38 |
|  | IMPHY004631 | Stearic acid | -5.98 | 32 |
|  | IMPHY004643 | Genistein | -5.66 | 44 |
|  | IMPHY004653 | Serotonin | -5.43 | 43 |
|  | IMPHY004660 | Luteolin | -5.58 | 30 |
|  | IMPHY006316 | N,N-Dimethyltryptamine | -4.59 | 65 |
|  | IMPHY006362 | Ascorbic acid | -4.07 | 20 |
|  | IMPHY007257 | Dopamine | -4.50 | 48 |
|  | IMPHY007301 | Nicotine | -4.36 | 57 |
|  | IMPHY007327 | Palmitic acid | -5.42 | 28 |
|  | IMPHY010037 | 6-methoxy-1-methyl-9H-pyrido[3,4-b]indole | -5.31 | 74 |
|  | IMPHY011394 | Arachidic acid | -5.48 | 24 |
|  | IMPHY011602 | 9H-Pyrido[3,4-B]indole | -4.85 | 72 |
|  | IMPHY011724 | (9Z)-(12S,13R)-12,13-Epoxyoctadecenoic acid | -6.18 | 13 |
|  | IMPHY011797 | Oleic acid | -6.28 | 33 |
|  | IMPHY011880 | Ursolic acid | -9.29 | 99 |
|  | IMPHY012003 | Betulinic acid | -9.32 | 65 |
|  | IMPHY012021 | Gallic acid | -5.49 | 70 |
|  | IMPHY012029 | Sterol | -6.74 | 98 |
|  | IMPHY012334 | Glutathione | -4.42 | 6 |
|  | IMPHY013742 | Levodopa | -5.48 | 26 |
|  | IMPHY014836 | beta-Sitosterol | -8.64 | 53 |
|  | IMPHY014842 | Stigmasterol | -8.50 | 28 |
|  | IMPHY014990 | Linoleic acid | -5.75 | 30 |

**S1 Fig.** Chemical structure of top 4 compounds


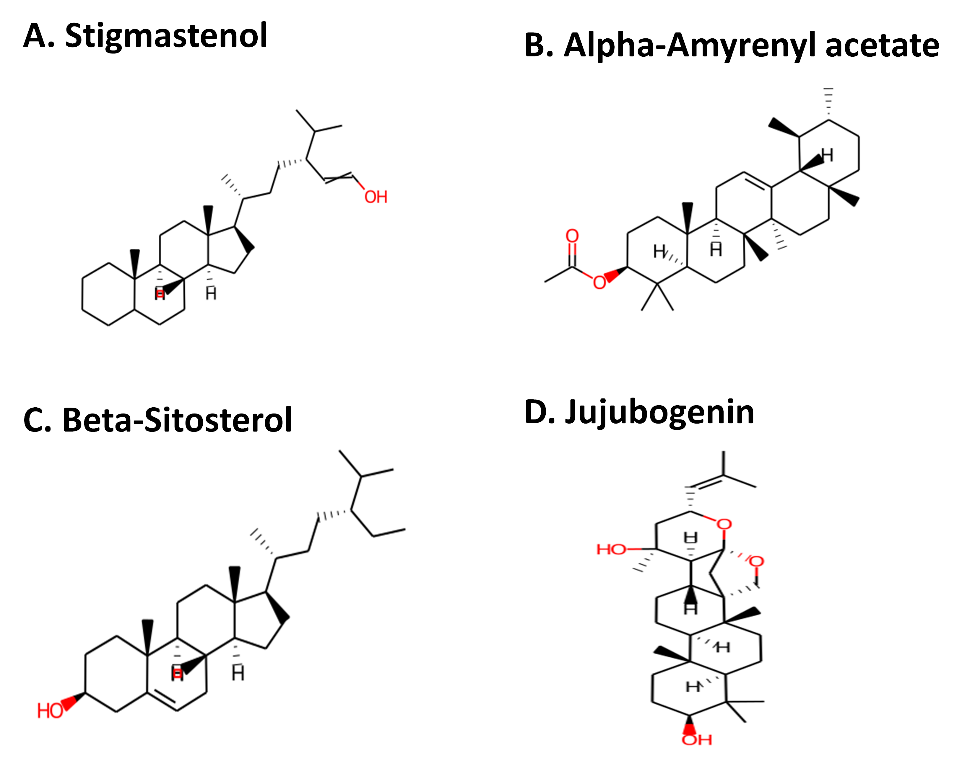


**Programming scripts**

**1. Molecular Docking**

**Grid script**

*autogrid4 -p grid.gpf -l grid.glg*

**Docking script**

*autodock4 -p docking.dpf -docking.dlg*

**2. Molecular Dynamics Simulation**

Gromax scripts for protein-ligand interaction (http://www.mdtutorials.com/gmx/complex/)

Trajectory analysis

A. For RMSD

*gmx rms -s MD.tpr -f MD_center.xtc -o rmsd.xvg -tu ns*

**B. For RMSF**

*gmx rmsf -f MD_center.xtc -s MD.tpr -o rmsf_res.xvg -res*

**C. For H-bond**

*gmx hbond -s MD.tpr -f MD_center.xtc -num hb.xvg -tu ns*

**D. For radius of gyration**

*gmx gyrate -s MD.tpr -f MD_center.xtc -o gyrate1.xvg*

**E. For SASA**

*gmx sasa -s MD.tpr -f MD_center.xtc -o sasa.xvg -tu ns*

**F. For PCA**

*gmx covar -f MD_center.xtc -s MD.tpr -n index.ndx -o eiginval.xvg -tu ns*

#Choose 3 for C-Alpha

#Choose 13 for Ligand

*gmx anaeig -v eigenvec.trr -f MD_center.xtc -s MD.tpr -n index.ndx -comp eigcomp.xvg -2d 2dproj.xvg -b 0 -tu ns -first 1 -last 3*

#Choose 3 for C-Alpha

#Choose 13 for Ligand

**E. For Free Energy Landscape**

*gmx sham -f PC1PC2.xvg -ls FES.xpm*

*python2.7 xpm2txt.py -f FES.xpm -o free-energy-landscape.dat*

**F. For protein-ligand pdb file**

*gmx trjconv -s MD.tpr -f MD_center.xtc -n index.ndx -o trj.pdb -b 0 -e 100 -skip 10*
