# Supplementary material for: Screening of phytoconstituents from Bacopa monnieri (L.) Pennell and Mucuna pruriens (L.) DC. to identify potential inhibitors against Cerebroside sulfotransferase
Source: PLoS One. 2024 Oct 24;19(10):e0307374. doi: 10.1371/journal.pone.0307374 (PMC11500956; doi:10.1371/journal.pone.0307374)
Supplement: Attachment — Submitted filename: Review report on Screening of phytoconstituents from Bacopa.pdf [file pone.0307374.s002.pdf]

The paper titled "Screening of phytoconstituents from Bacopa monnieri (L.) Pennell and Mucuna pruriens (L.) DC. to identify potential inhibitors against Cerebroside Sulfotransferase" presents a comprehensive computational study aimed at identifying potential inhibitors against Cerebroside Sulfotransferase (CST) as a therapeutic target for metachromatic leukodystrophy (MLD). The study employs a multistep virtual screening approach, molecular dynamics simulations, and pharmacokinetic analyses to identify four phytoconstituents with potential inhibitory activity against CST. The paper is well-structured and provides detailed insights into each step of the computational methodology, along with thorough results and discussion sections.

- The introduction provides a thorough overview of MLD, its pathophysiology, and current therapeutic approaches, which contextualizes the significance of identifying CST inhibitors. However, it would be beneficial to provide more context on the existing challenges and limitations in developing CST inhibitors, particularly focusing on the lack of experimental structural data for CST, which necessitates the use of computational modeling and virtual screening approaches.

- The methodology section is comprehensive and well-described. It provides sufficient details on protein preparation, ligand screening, molecular docking, pharmacokinetic analyses, MM-PB/GBSA calculations, molecular dynamics simulations, and cross-target identification. However, it would be helpful to include the programming script used for each step of the computational analysis, as this can impact reproducibility and compatibility with future studies.

- The results section presents a detailed analysis of the screening process, including the identification of top hits based on binding scores, pharmacokinetic properties, MM-PB/GBSA calculations, molecular dynamics simulations, and cross-target identification.

- The discussion section effectively interprets the findings in the context of the study objectives and provides insights into the potential implications of the identified inhibitors for MLD therapy. However, it would be valuable to discuss the limitations of the computational approach employed, including assumptions and uncertainties associated with virtual screening, molecular dynamics simulations, and pharmacokinetic predictions.

- Additionally, discussing the potential experimental validation of the identified inhibitors, such as in vitro enzymatic assays or cell-based assays, would strengthen the discussion and provide direction for future research.

- The conclusion provides a concise summary of the study findings and emphasizes the potential of the identified phytoconstituents as CST inhibitors for MLD therapy. It might be beneficial to include recommendations for future research directions, such as experimental validation studies or further optimization of the identified inhibitors.

- Ensure the manuscript is free of typographical and grammatical errors. Proofreading and editing will enhance readability.

- Overall, the paper presents a rigorous computational study with well-defined methodologies and comprehensive analyses. The findings contribute to the understanding of CST inhibition for MLD therapy and offer potential drug candidates for further investigation.

- Addressing minor points such as providing additional context, discussing limitations, and suggesting future research directions would enhance the clarity and impact of the paper. I recommend accepting the manuscript after these minor revisions are made.
