# Supplementary material for: Screening of phytoconstituents from Bacopa monnieri (L.) Pennell and Mucuna pruriens (L.) DC. to identify potential inhibitors against Cerebroside sulfotransferase
Source: PLoS One. 2024 Oct 24;19(10):e0307374. doi: 10.1371/journal.pone.0307374 (PMC11500956; doi:10.1371/journal.pone.0307374)
Supplement: Attachment — Submitted filename: Response to reviewers.docx [file pone.0307374.s003.docx]

**Reply to Editor:**

1. Please ensure that your manuscript meets PLOS ONE's style requirements, including those for file naming. The PLOS ONE style templates can be found at

<https://journals.plos.org/plosone/s/file?id=wjVg/PLOSOne_formatting_sample_main_body.pdf>

and

<https://journals.plos.org/plosone/s/file?id=ba62/PLOSOne_formatting_sample_title_authors_affiliations.pdf>

**Reply**: We have modified the manuscript as per Plos One style.

2. Thank you for stating the following financial disclosure:

"Institute of Eminence, Banaras Hindu University, Government of India

Grant ID: R/Dev/G/6031/IoE/MPDFs/61698"

Please state what role the funders took in the study. If the funders had no role, please state: "The funders had no role in study design, data collection and analysis, decision to publish, or preparation of the manuscript." If this statement is not correct you must amend it as needed.

Please include this amended Role of Funder statement in your cover letter; we will change the online submission form on your behalf.

Reply: “The funder has role in providing me post-doctoral fellowship and computing facilities and other infrastructure for this research.”

3. Please note that funding information should not appear in the Acknowledgments section or other areas of your manuscript. We will only publish funding information present in the Funding Statement section of the online submission form. Please remove any funding-related text from the manuscript.

**Reply**: Funding information is removed from the main manuscript.

4. We note that Figure 1 in your submission contains copyrighted images. All PLOS content is published under the Creative Commons Attribution License (CC BY 4.0), which means that the manuscript, images, and Supporting Information files will be freely available online, and any third party is permitted to access, download, copy, distribute, and use these materials in any way, even commercially, with proper attribution. For more information, see our copyright guidelines: <http://journals.plos.org/plosone/s/licenses-and-copyright>.

**Reply**: Figure 1 is exclusively designed by us to summarize the entire work presented in this manuscript. This figure is not a copyrighted image. If you find a similar figure, please let me know. I have further modified the figure. Please let me know, still it is a case of copyright infringement?

5. We require you to either (1) present written permission from the copyright holder to publish these figures specifically under the CC BY 4.0 license, or (2) remove the figures from your submission:

**Reply**: Figure 1 is now removed

1) You may seek permission from the original copyright holder of Figure 1 to publish the content specifically under the CC BY 4.0 license.

We recommend that you contact the original copyright holder with the Content Permission Form (http://journals.plos.org/plosone/s/file?id=7c09/content-permission-form.pdf) and the following text:

“I request permission for the open-access journal PLOS ONE to publish XXX under the Creative Commons Attribution License (CCAL) CC BY 4.0 (http://creativecommons.org/licenses/by/4.0/). Please be aware that this license allows unrestricted use and distribution, even commercially, by third parties. Please reply and provide explicit written permission to publish XXX under a CC BY license and complete the attached form.”

Please upload the completed Content Permission Form or other proof of granted permissions as an ""Other"" file with your submission.

In the figure caption of the copyrighted figure, please include the following text: “Reprinted from [ref] under a CC BY license, with permission from [name of publisher], original copyright [original copyright year].”

2) If you are unable to obtain permission from the original copyright holder to publish these figures under the CC BY 4.0 license or if the copyright holder’s requirements are incompatible with the CC BY 4.0 license, please either i) remove the figure or ii) supply a replacement figure that complies with the CC BY 4.0 license. Please check copyright information on all replacement figures and update the figure caption with source information.

If applicable, please specify in the figure caption text when a figure is similar but not identical to the original image and is therefore for illustrative purposes only.

**Reply**: Since Figure 1 is remove, no issue with copyright is applicable now.

5. Please include captions for your Supporting Information files at the end of your manuscript, and update any in-text citations to match accordingly. Please see our Supporting Information guidelines for more information: <http://journals.plos.org/plosone/s/supporting-information>.

**Reply**: Captions for Supporting Information file is included at the end of the manuscript.

**Additional Editor Comments:**

The manuscript entitled “Screening of phytoconstituents from Bacopa monnieri (L.) Pennell and Mucuna pruriens (L.) DC. to identify potential inhibitors against Cerebroside Sulfotransferase” has undergone extensive review. Based on the reviewers' comments and suggestions, the authors need to revise the manuscript thoroughly to enhance its quality and readability.

**Reply**: We followed the suggestion of all three respected reviewers to modify the manuscript and proofread multiple times to enhance the readability of the manuscript.

**Reply to Reviewers**

Reply to Reviewer #1:

1. Check all full stops placed before and after reference citations.

**Reply**: We are thankful for reviewer to keenly review the manuscript and highlighted the reference part. References are cited as per reviewer’s comment.

2. Better to write all amino acids with their position as Lys82 rather than LYS82.

**Reply**: Modified the amino acids representation as per the suggestion.

3. Check all abbreviations such as TPSA, HBA, HBD, and others not defined.

**Reply**: Abbreviations are defined now and rechecked.

4. What is meaning of Predicted toxicity class 1-6? (which one is good, better, best, or worst?)

**Reply**: ProTox divides the compounds into different toxicity groups (classes) based on the severity of their effect. As explained here, web server ProTox uses the GHS toxicity classification whereby compounds are divided into 6 classes – 1-5 classes represent different grades of toxicities and class 6 is mostly the non-toxic class. 1 represented in red is highly toxic while 6 represented in green is non-toxic. Compounds fall in class 4 or above are considered within the acceptable limit of toxicity.

5. In Table 3, column interaction type and distance not represented a well and meaningful way. Instead of these columns, add a column H-bonding residues.

**Reply**: The interaction type column includes all possible interactions including hydrogen bonds and all other interactions and based on that interaction types, the result text part is written in elaborated manner. Distances of these bonds suggest the strength of each bonding for over all binding and deciding the orientation of compounds in the active site.

6. In the abstract, the sentence "These compounds bound to the active site pocket of CST by interacting with LYS82, LYS85, SER89, TYR176, PHE170, PHE177 in the binding pocket." Say something but residues in table 3 indicate something different. Check and revise.

**Reply**: We are thankful for reviewer to highlighting this error. The sentence is corrected and revised now.

7. Check capital and small case. use a uniform style. case: upper panel Lower panel

**Reply**: Rechecked and corrected where the uniformity was missing.

8. Check this sentence "The average intramolecular hydrogen bonds for free CST, CST-GC, IMPHY001534, CST-IMPHY004141, CST-IMPHY009537, and IMPHY014836 were 175.37, 186.13, 172.20, 180.62, 174.09, and 175.8, respectively"

**Reply**: We have rechecked the sentence. It is all right.

9. It will be better to use a compound name rather than an ID as there exists a different ID for the same compound in different chemical databases.

**Reply**: Now all the selected compounds are denoted with compound names throughout the manuscript.

10. In Table 5, interaction type and distance are not represented in a meaningful way. Instead of these columns, add a column H-bonding residues.

**Reply**: Since the interaction type includes all kinds of weak interactions between ligand and protein including hydrogen bonds, pi-pi, pi-alkyl, pi-sigma, Vander Waal, etc. The distance in Å represents the length of the bonds. Beside hydrogen bonding the ligand interact via various other interactions which also played critical role in deciding orientation and hold of the compounds in the active site of proteins.

11. There are many more figures, and many subfigures placed on a figure. I suggest you to place some figures as supplementary. Also, improve the quality of text and clarity of figures.

**Reply**: In this manuscript total nine figures present. Figure 1 of that represented the workflow is now removed. The present Figure 1 and Figure 7 highlighted the interaction pattern in surface view, 3D views and 2D view in pre and postMD, respectively. Figure 1 (which was earlier Figure 2) is now more simplified. Figure 2 to Figure 4 represent various validations of trajectory while Figure 5 and 6 represents PCA and FEL studies, respectively. The contents in all these figures are discussed elaborated manner on result section. If we shift them in supplementary file, it would restraint the readability. The reason for multiple subfigures is that all figures contain comparative result of all four top hits. Nevertheless, if reviewer still find it not suitable for main manuscript, please suggest precisely which figure need to move to supplementary file.

**Reply to Reviewer #2:**

1. The paper titled "Screening of phytoconstituents from Bacopa monnieri (L.) Pennell and Mucuna pruriens (L.) DC. to identify potential inhibitors against Cerebroside Sulfotransferase" presents a comprehensive computational study aimed at identifying potential inhibitors against Cerebroside Sulfotransferase (CST) as a therapeutic target for metachromatic leukodystrophy (MLD). The study employs a multistep virtual screening approach, molecular dynamics simulations, and pharmacokinetic analyses to identify four phytoconstituents with potential inhibitory activity against CST. The paper is well-structured and provides detailed insights into each step of the computational methodology, along with thorough results and discussion sections.

**Reply**: We are thankful to reviewer for positively summarize the entire work.

2. The introduction provides a thorough overview of MLD, its pathophysiology, and current therapeutic approaches, which contextualizes the significance of identifying CST inhibitors. However, it would be beneficial to provide more context on the existing challenges and limitations in developing CST inhibitors, particularly focusing on the lack of experimental structural data for CST, which necessitates the use of computational modeling and virtual screening approaches.

**Reply:** Thank you for pointing out this important upgradation. As per your suggestion I have concisely written the challenges and limitation part in Introduction and highlighted in yellow.

3. The methodology section is comprehensive and well-described. It provides sufficient details on protein preparation, ligand screening, molecular docking, pharmacokinetic analyses, MM-PB/GBSA calculations, molecular dynamics simulations, and cross-target identification. However, it would be helpful to include the programming script used for each step of the computational analysis, as this can impact reproducibility and compatibility with future studies.

**Reply:** Programming script in detail is provided in supplementary section.

4. The results section presents a detailed analysis of the screening process, including the identification of top hits based on binding scores, pharmacokinetic properties, MM-PB/GBSA calculations, molecular dynamics simulations, and cross-target identification.

**Reply:** We are very much grateful for reviewer for his positive remark on the result section.

5. The discussion section effectively interprets the findings in the context of the study objectives and provides insights into the potential implications of the identified inhibitors for MLD therapy. However, it would be valuable to discuss the limitations of the computational approach employed, including assumptions and uncertainties associated with virtual screening, molecular dynamics simulations, and pharmacokinetic predictions.

**Reply**: In the last para of discussion, we have discussed the limitations of computational approach along with some way forwards

6. Additionally, discussing the potential experimental validation of the identified inhibitors, such as in vitro enzymatic assays or cell-based assays, would strengthen the discussion and provide direction for future research.

**Reply**: In the second last and last para of discussion we have discussed on potential experimental validation of the identified inhibitors and future directions.

7. The conclusion provides a concise summary of the study findings and emphasizes the potential of the identified phytoconstituents as CST inhibitors for MLD therapy. It might be beneficial to include recommendations for future research directions, such as experimental validation studies or further optimization of the identified inhibitors.

**Reply:** As per the suggestion in conclusion part we have included future direction. The changes are highlighted in yellow colour.

8. Ensure the manuscript is free of typographical and grammatical errors. Proofreading and editing will enhance readability.

**Reply:** The manuscript is proofread carefully for multiple times. We hope, now manuscript is in good shape.

9. Overall, the paper presents a rigorous computational study with well-defined methodologies and comprehensive analyses. The findings contribute to the understanding of CST inhibition for MLD therapy and offer potential drug candidates for further investigation.

**Reply:** We are sincerely grateful to the reviewer for his encouraging comments on the manuscript writing part and work done for this manuscript.

10. Addressing minor points such as providing additional context, discussing limitations, and suggesting future research directions would enhance the clarity and impact of the paper. I recommend accepting the manuscript after these minor revisions are made.

**Reply:** We are very much grateful to reviewer for his recommendation for acceptance of the manuscript. We have carefully addressed all comments and revised the manuscript.

**Reply to Reviewer #3:**

The present paper describes screening of phytoconstituents from Bacopa monnieri (L.) Pennell and Mucuna pruriens (L.) DC. to identify potential inhibitors against Cerebroside Sulfotransferase. The research is well documented and presented through docking analysis and they have identified IMPHY009537 from Bacopa monnieri IMPHY004141 from Mucuna pruriens as the second-best performing inhibitor against CST. The studies are important for development of oral drug for inhibiting CST and to inhibit the metachromatic leukodystrophy disease.

**Reply:** We are thankful for reviewer for such a positive comments and support for acceptance of manuscript.
